# Shared Decision-Making and Emergency Department Use Among People With High Blood Pressure

**DOI:** 10.5888/pcd20.230086

**Published:** 2023-09-21

**Authors:** R. Aver Yakubu, Alyssa Coleman, Alina Ainyette, Anisha Katyayan, Kimberly R. Enard

**Affiliations:** 1 Department of Health Management and Policy, College for Public Health and Social Justice, Saint Louis University, St. Louis, Missouri

## Abstract

**Introduction:**

Forty-seven percent of all adults in the US have a diagnosis of high blood pressure. Among all US emergency department (ED) users, an estimated 45% have high blood pressure. The success of high blood pressure interventions in reducing ED visits is partially predicated on patients’ adherence to treatment plans. One method for promoting adherence to treatment plans is shared decision-making between patients and medical providers.

**Methods:**

We conducted a cross-sectional observational study using 2015–2019 Medical Expenditure Panel Survey data. We used studies on shared decision-making as a guide to create a predictor variable for shared decision-making. We determined covariates according to the Andersen Behavioral Model of Health Services Use. ED use was the outcome variable. We used cross tabulation to compare covariates of ED use and multivariable logistical regression to assess the association between shared decision-making and ED use. Our sample size was 30,407 adults.

**Results:**

Less than half (39.3%) of respondents reported a high level of shared decision-making; 23.3% had 1 or more ED visits. In the unadjusted model, respondents who reported a high level of shared decision-making were 20% less likely than those with a low level of shared decision-making to report 1 or more ED visits (odds ratio [OR], 0.80; 95% CI, 0.75–0.86; *P* <.001). After adjusting for covariates, a high level of shared decision-making was still associated with lower odds of ED use (OR, 0.86; 95% CI, 0.76–0.97; *P* = .01).

**Conclusion:**

Shared decision-making may be an effective method for reducing ED use among patients with high blood pressure.

**Summary**

**What is already known on this topic?**

Several studies have assessed the relationship among chronic disease, shared decision-making, and health care use, but information is limited on the relationship among high blood pressure, shared decision-making, and use of emergency department services.

**What is added by this report?**

Findings from this report provide insight on how predisposing, enabling, and need factors based on the Andersen model contribute to shared decision-making and emergency department use.

**What are the implications for public health practice?**

Future studies can expand on the perceived use of shared decision-making among people with chronic disease to improve outcomes and types of health care services used.

## Introduction

Heart disease is the leading cause of death in the US and worldwide (1). In the US, 47% of all adults have been diagnosed with one of the major risk factors for heart disease, high blood pressure (2). High blood pressure is often called the silent killer because many people are asymptomatic and unaware of their condition (3). Among all adults diagnosed with high blood pressure, only 1 in 4 have their high blood pressure under control (able to lower blood pressure with medication) (2). Uncontrolled high blood pressure is associated with increased risk for preventable emergency department (ED) visits. From 2006 to 2012, in the most recent analysis available on ED use and high blood pressure, ED visits caused by high blood pressure increased by 4% each year (4). Among all ED users in the US, an estimated 45% have high blood pressure (5). The COVID-19 pandemic further exacerbated these trends, with systolic blood pressure increasing on average by 1.79 mm Hg and diastolic blood pressure increasing on average by 1.30 mm Hg from the prepandemic period (August 2018 through January 2020) to the pandemic period (April 2020 through November 2020) (6). Experts have recommended evidence-based interventions for reducing high blood pressure as a way to save 100 million lives worldwide by 2040 (7). Reductions in the use of EDs for managing high blood pressure can serve as a proxy for the successful implementation of interventions to reduce high blood pressure. However, the success of high blood pressure interventions and reducing ED visits is partially predicated on patients adhering to treatment plans. One method for promoting adherence to treatment plans is a positive relationship between a patient and a clinician (8). 

Positive patient–clinician relationships improve patient satisfaction, medication adherence, and successful development of treatment plans (9–12). A component of patient–provider relationships is shared decision-making. Shared decision-making is a collaboration in which treatment options are explained by the clinician and the patient provides feedback on what they prefer (13). Shared decision-making is achieved when patients are empowered to be involved in all aspects of health care discussions and decision-making (14). Several studies have assessed the relationship among chronic disease, shared decision-making, and health care use (10,15,16). However, knowledge is limited on the relationship among high blood pressure, shared decision-making, and ED use (4,7,10,17).

Previous studies indicate that the Andersen Behavioral Model of Health Services Use, often referred to as the Andersen model, is an appropriate framework for assessing behaviors contributing to health care use and shared decision-making (18,19). The Andersen model has various iterations, but the fundamental components are predisposing factors, enabling factors, and need factors (18,19). Predisposing factors are individual characteristics that would influence a person toward use of health care; examples are age, education, race, and ethnicity. Enabling factors are external resources that create the ability to use care; examples are transportation, health insurance, and the ability to pay for health care. Having access to a clinician who engages in shared decision-making could also be considered an enabling factor. Need factors are the individual’s or clinician’s perception of whether the individual needs care. According to the Andersen model, people are more inclined to seek health care when they perceive a greater need for care, have access to enabling resources, and possess predisposing factors that motivate them to seek care. Studies on shared decision-making and the Andersen model posit that shared decision-making improves equity in care and supports positive behavior in the use of health care services, such as seeking preventive and primary care services rather than ED services to manage chronic conditions (20). This model proves valuable in comprehending the intricate interactions among these factors, thereby aiding in the analysis of patterns in health care use across diverse populations.

The objective of this study was to assess the relationship between shared decision-making and ED use among adults with a diagnosis of high blood pressure. The Andersen model provided a framework for our study to explain how ED use is influenced by these predisposing factors, enabling factors, and need factors. We hypothesized that a high level of shared decision-making would be significantly associated with lower levels of ED use.

## Methods

### Study design

We conducted a cross-sectional observational study using 2015–2019 Medical Expenditure Panel Survey (MEPS) data, a population-based survey managed by the Agency for Healthcare Research and Quality (21). MEPS collects data by using a set of large-scale surveys of families and individuals, their clinician, and employers across the US. Collectively, these data offer a nationally representative sample of the US population. Our study used data from the household component, which draws from a subsample of households that participated in the previous year’s National Health Interview Survey (administered by the National Center for Health Statistics). The panel design includes several rounds of interviews that cover 2 calendar years to assess changes in health status, income, employment, and use of services. Inclusion criteria for our study were being aged 18 years or older (n = 118,839), having ever been diagnosed with high blood pressure (n = 40,605), and having attended at least 1 physician’s visit in the previous year (n = 51,992); 30,407 respondents met all 3 criteria and were included in our final analytic sample. Respondents with missing data for any of the 3 inclusion criteria were excluded from the final analytic sample. The Saint Louis University Institutional Review Board (IRB) determined that this study was exempt from IRB review.

### Variables

#### Predictor variable: shared decision-making

On the basis of previous SDM-related studies (10,15), we developed a predictor variable for SDM by averaging 7 MEPS questions into a single composite score. The 7 questions were as follows:

- If there were a choice between treatments, how often would your medical provider ask you to help make the decision?
- Does a medical person at your usual source of care present and explain all options to you?
- Thinking about the types of medical, traditional, and alternative treatments you are happy with, how often does your medical provider show respect for these treatments?
- In the last 12 months, how often did your doctors or other health providers listen carefully to you?
- In the last 12 months, how often did your doctors or other health providers explain things in a way that you could understand?
- In the last 12 months, how often did your doctors or other health providers show respect for what you had to say?
- In the last 12 months, how often did your doctors or other health providers spend enough time with you?

Six of the shared decision-making questions were on a 4-point Likert scale ranging from 1 (never) to 4 (always). The remaining shared decision-making question (Does a medical person at your usual source of care present and explain all options to you?) had a yes/no response. We recoded this response as 1 (no) or 4 (yes). We recoded the mean values of the shared decision-making composite scores to a binary variable: low level of shared decision-making (mean summary score <3.9) and a high level of shared decision-making (mean summary score ≥3.9). This method was successfully tested for validity by Lindly et al (22).

#### Outcome variable: ED use

We used a single MEPS item for number of ED visits to create a variable for ED use. We recoded this variable as a binary variable: 1 or more ED visits versus 0 ED visits.

#### Covariates

We determined covariates on the basis of applicable predisposing, enabling, and need factors of the Andersen model available in the MEPS data set. Predisposing factors were age, sex, race and ethnicity, geographic region, highest educational degree earned, body mass index (BMI), and personal belief about seeing a physician. For this last item, we used responses to the MEPS question on respondents believing they can “overcome ills without medical help,” which we categorized as “uncertain or disagree” or “agree.” Enabling factors were income, based on the poverty category variable in MEPS (high [>400% poverty line]), middle [200%–400% poverty line], and low [<200% poverty line]), travel time to a doctor’s appointment, and health insurance (any private, public only, uninsured). For need factors, we considered only 1 variable to be applicable: self-perceived general health status. All covariates were categorical variables.

### Statistical analysis

We used Stata version 14 MP (StataCorp LLC) to conduct all statistical analyses. We used appropriate sample weights to account for the complex survey design and produce nationally representative prevalence estimates. A descriptive overview of the sample included counts and percentages. We used χ^2^ tests to assess significant relationships in cross tabulations between each variable and the outcome (ED use). We used binary logistic regression to assess associations between shared decision-making and ED use with covariates. Significance was set at *P *≤.05. 

## Results

Of the final sample, less than half (39.3%) of respondents reported a high level of shared decision-making; 23.3% had 1 or more ED visits (Table 1). Most patients were aged 40 years or older (40–64 y, 46.2%; ≥65 y, 44.3%). By sex, we found an almost even distribution of men (49.3%) and women (50.7%). Most (68.7%) respondents were White only. The highest educational degree earned among most (54.7%) of respondents was a high school diploma or GED; 79.9% were classified as obese based on BMI, 42.5% had a high income, 63.4% had private insurance, and most considered themselves to have good (39.9%) or very good (28.4%) health status.

**Table 1:** Descriptive Statistics of Final Analytic Sample in Study on Shared Decision-Making and ED Use Among Adults Aged ≥18 Years With a Diagnosis of High Blood Pressure, US, 2015−2019^a^

Variable	No. (%)
**Total**	30,407 (100.0)
**Level of shared decision-making^b^**	
Low	18,357 (60.7)
High	12,050 (39.3)
**No. of visits to ED**	
0	23,037 (76.7)
≥1	7,370 (23.3)
**Predisposing factors^c^**	
Age, y	
18–39	2,751 (9.5)
40–64	13,958 (46.2)
≥65	13,600 (44.3)
Sex	
Male	14,117 (49.3)
Female	16,290 (50.7)
Race and ethnicity	
Asian only	1,384 (4.1)
Black only	6,237 (13.7)
Hispanic	5,033 (10.5)
White only	16,832 (68.7)
Other or multiple races	921 (3.0)
Geographic region	
Northeast	4,879 (16.7)
Midwest	6,258 (21.8)
South	12,629 (40.9)
West	6,544 (20.5)
Highest educational degree earned	
No degree	4,822 (14.0)
High school diploma or GED	13,527 (54.7)
Bachelor’s	4,087 (18.8)
Master’s or doctorate	2,642 (12.5)
Body mass index^d^	
Underweight (<18.5)	361 (1.2)
Normal (18.5 to <25.0)	2,118 (7.1)
Overweight (25.0 to <30.0)	3,518 (11.8)
Obese (≥30.0)	24,342 (79.9)
Personal belief in ability to “overcome ills without medical help”	
Disagree/uncertain	19,307 (86.7)
Agree	2,716 (13.3)
**Enabling factors^c^**	
Income	
High (>400% poverty line)	8,200 (42.5)
Middle (200%–400% poverty line)	7,024 (27.5)
Low (<200% poverty line)	9,743 (30.0)
Travel time to doctor’s appointment, min	
<15	13,378 (50.0)
15–30	10,559 (38.6)
31–60	2,733 (9.6)
>61	526 (1.8)
Health insurance	
Any private	17,001 (63.4)
Public only	12,116 (33.3)
Uninsured	1,290 (3.3)
**Need factor^c^**	
Self-perceived general health status	
Excellent	1,798 (6.9)
Very good	7,347 (28.4)
Good	11,411 (39.9)
Fair	6,549 (19.7)
Poor	1,689 (5.1)

Abbreviation: ED, emergency department; GED, General Educational Development.

a Data source: 2015–2019 Medical Expenditure Panel Survey (MEPS) (21). All data are weighted; percentages may not add to 100 because of rounding.

b Answers to 7 MEPS questions related to shared decision-making were used to develop a predictor variable for shared decision-making.

c The Andersen model (18,19) was used as a framework to explain how ED use was influenced by predisposing factors, enabling factors, and need factors, the 3 fundamental components of this model. Only 1 need factor applied to the MEPS data set.

d Calculated as body weight in kilograms divided by height in meters squared.

### Shared decision-making and ED use

The independent variable and all covariates were significantly associated with ED use (Table 2). Among the Asian-only group, 11.5% reported 1 or more ED visits; 20% or more of all other racial and ethnic groups reported 1 or more ED visits. In unadjusted models of the association between shared decision-making and ED use, respondents who reported a high level of shared decision-making were 20% less likely than respondents who reported a low level of shared decision-making to report 1 or more ED visits (OR, 0.80; 95% CI, 0.75–0.86; *P* < .001) (Table 3). After adjusting for covariates in the model, a high level of shared decision-making was still associated with lower odds of ED use: respondents with a high level of shared decision-making were 14% less likely to report 1 or more ED visits (OR, 0.86; 95% CI, 0.76–0.97; *P* = .01).

**Table 2:** Cross-Tabulation Analysis Between Variables and Outcome (ED Use) in Study on Shared Decision-Making and ED Use Among Adults Aged ≥18 Years With a Diagnosis of High Blood Pressure, US, 2015−2019^a^

Variable	No. of visits to ED, %		*P* value^b^
	No ED visit, %	≥1 ED visits	
**Level of shared decision-making^c^**			
Low	75.2	24.8	<.001
High	79.0	21.0	
**Predisposing factors^d^**			
Age, y			
18–39	76.7	23.3	<.001
40–64	79.0	21.0	
≥65	74.6	25.5	
Sex			
Male	79.6	20.4	<.001
Female	73.9	26.1	
Race and ethnicity			
Asian only	88.5	11.5	<.001
Black only	73.4	26.6	
Hispanic	78.5	21.5	
White only	76.8	23.2	
Other or multiple races	68.1	31.9	
Geographic region			
Northeast	76.5	23.5	.02
Midwest	75.5	24.6	
South	76.7	23.4	
West	78.8	21.2	
Highest educational degree earned			
No degree	69.9	30.1	<.001
High school diploma/GED	74.8	25.2	
Bachelor’s	83.0	17.0	
Master’s or doctorate	82.0	18.0	
Body mass index^e^			
Underweight (<18.5)	73.3	26.7	.03
Normal (18.5 to <25.0)	76.8	23.2	
Overweight (25.0 to <30.0)	79.1	20.9	
Obese (≥30.0)	76.4	23.6	
Personal belief in ability to “overcome ills without medical help”			
Disagree or uncertain	76.0	24.0	<.001
Agree	80.7	19.3	
**Enabling factors^d^**			
Income			
High (>400% poverty line)	82.5	17.5	<.001
Middle (200%–400% poverty line)	77.1	22.9	
Low (<200% poverty line)	69.3	30.7	
Travel time to doctor’s appointment, min			
<15	77.2	22.8	<.001
15–30	76.7	23.3	
31–60	72.7	27.3	
>61	77.4	22.7	
Health insurance			
Any private	80.6	19.5	<.001
Public only	69.2	30.8	
Uninsured	78.6	21.5	
**Need factor^d^**			
Self-perceived general health status			
Excellent	88.9	11.1	<.001
Very good	85.6	14.4	
Good	77.7	22.3	
Fair	64.8	35.2	
Poor	51.8	48.2	

Abbreviation: ED, emergency department; GED, General Educational Development.

a Data source: 2015–2019 Medical Expenditure Panel Survey (MEPS) (21). All data are weighted.

b Determined by Pearson χ^2^ test; *P* ≤.05 considered significant.

c Answers to 7 MEPS questions related to shared decision-making were used to develop a predictor variable for shared decision-making.

d The Andersen model (18,19) was used as a framework to explain how ED use was influenced by predisposing factors, enabling factors, and need factors, the 3 fundamental components of this model. Only 1 need factor applied to the MEPS data set.

e Calculated as body weight in kilograms divided by height in meters squared.

**Table 3:** Association Between Shared Decision-Making and ED Use in Study on Shared Decision-Making and ED Use Among Adults Aged ≥18 With a Diagnosis of High Blood Pressure, US, 2015−2019^a^

Variable	Odds ratio (95% CI)	*P* value^b^
**High level of shared decision-making^c^**		
Unadjusted	0.80 (0.75–0.86)	<.001
Adjusted	0.86 (0.76–0.97)	.01
**Predisposing factors^d^**		
Age, y		
18–39	1 [Reference]	
40–64	0.76 (0.62–0.93)	.007
≥65	0.94 (0.77–1.14)	.51
Sex		
Male	1 [Reference]	
Female	1.26 (1.11–1.43)	<.001
Race and ethnicity		
Asian only	0.42 (0.29–0.60)	<.001
Black only	1.01 (1.00–1.54)	.95
Hispanic	0.81 (0.66–0.99)	.04
White only	1 [Reference]	
Other or multiple races	1.36 (1.00–1.86)	.052
Geographic region		
Northeast	1 [Reference]	
Midwest	1.03 (0.87–1.22)	.75
South	0.90 (0.77–1.05)	.20
West	0.93 (0.78–1.11)	.43
Highest educational degree earned		
No degree	1 [Reference]	
High school diploma/GED	1.02 (0.85–1.23)	.81
Bachelor’s	0.92 (0.72–1.18)	.52
Master’s or doctorate	1.03 (0.81–1.32)	.80
Body mass index^e^		
Underweight (<18.5)	1 [Reference]	
Normal (18.5 to <25.0)	1.07 (0.76–1.50)	.71
Overweight (25.0 to <30.0)	0.94 (0.67–1.29)	.70
Obese (≥30.0)	1.06 (0.78–1.46)	.70
Personal belief in ability to “overcome ills without medical help”		
Disagree/uncertain	1 [Reference]	
Agree	0.89 (0.75–1.06)	.19
**Enabling factors^d^**		
Income		
High (>400% poverty line)	1 [Reference]	
Middle (200%–400% poverty line)	1.14 (0.97–1.33)	.11
Low (<200% poverty line)	1.35 (1.16–1.58)	<.001
Travel time to doctor’s appointment, min		
<15	1 [Reference]	
15–30	1.01 (0.89–1.13)	.93
31–60	1.27 (1.06–1.53)	.01
>61	0.80 (0.50–1.26)	.33
Health insurance		
Any private	1 [Reference]	
Public only	1.22 (1.06–1.40)	.004
Uninsured	1.09 (0.77–1.53)	.64
**Need factor^d^**		
Self-perceived general health status		
Excellent	1 [Reference]	
Very good	1.31 (0.97–1.76)	.08
Good	1.91 (1.44–2.51)	<.001
Fair	3.39 (2.58–4.47)	<.001
Poor	5.44 (3.78–7.83)	<.001

Abbreviation: ED, emergency department; GED, General Educational Development.

a Data source: 2015–2019 Medical Expenditure Panel Survey (MEPS) (21). All data are weighted.

b *P* ≤.05 considered significant.

c Answers to 7 MEPS questions related to shared decision-making were used to develop a predictor variable for shared decision-making.

d The Andersen model (18,19) was used as a framework to explain how ED use was influenced by predisposing factors, enabling factors, and need factors, the 3 fundamental components of this model. Only 1 need factor applied to the MEPS data set.

e Calculated as body weight in kilograms divided by height in meters squared.

### Andersen model covariates and ED use

The highest prevalence of having 1 or more ED visits occurred among respondents who had no educational degree (30.1%), public-only insurance (30.8%), or low income (30.7%). The percentage of respondents who had 1 or more ED visits was higher among respondents who disagreed or were uncertain they could overcome ills without medical help than among respondents who agreed they could overcome ills on their own (24.0% vs 19.3%) (Table 2).

Among predisposing factors, several categories of age, sex, and race and ethnicity were significantly associated with having 1 or more ED visits (Table 3). The odds of having 1 or more ED visits were 24% lower among respondents aged 40 to 64 years than among respondents 18 to 39 years (OR, 0.76; 95% CI, 0.62–0.93; *P* = .007). Women had a 26% higher likelihood of ED use than men (OR, 1.26; 95% CI, 1.11–1.43; *P* <.001). Compared with the White-only group, the Asian-only group had 58% lower odds (OR, 0.42; 95% CI, 0.29–0.60; *P* <.001) and the Hispanic group had 19% lower odds (OR, 0.81; 95% CI, 0.66–0.99; *P* = .04) of ED use. The odds of ED use were similar for the Black and White groups. The following enabling factors were significantly associated with ED use: low income, 31 to 60 minutes of travel time to a doctor’s appointment, and public-only insurance. Respondents with low income were 35% more likely than respondents with high income to use the ED (OR, 1.35; 95% CI, 1.16–1.58; *P* <.001). Having a longer travel time to doctor’s appointment (31–60 min vs <15 min) was also associated with higher odds of ED use (OR, 1.27; 95% CI, 1.06–1.53; *P* = .01). Respondents with public-only insurance were 22% more likely than respondents with private insurance to use the ED (OR, 1.22; 95% CI, 1.06–1.40; *P* = .004). For self-perceived health status, respondents with poor health status were 5.44 times more likely than respondents with excellent self-perceived health status to have a high level of shared decision-making (OR, 5.44; 95% CI, 3.78–7.83; *P* = <.001).

## Discussion

In our study, we used the Andersen model as a framework to assess the relationship between shared decision-making and ED use among patients with a high blood pressure diagnosis. Like other related studies, our study showed that less than half of patients reported a high level of shared decision-making, yet a high level of shared decision-making was associated with lower odds of ED use (16,22). Female sex and having low income, public-only insurance, or poor perceived health status were associated with higher odds of ED use.

We found several predisposing factors that contributed to increased ED use and differences in perceived shared decision-making. For example, among those who reported a high level of shared decision-making, women had higher odds than men of ED use. Findings in other studies on sex and ED use varied in terms of which sex had greater rates of ED use. In a study of women and men with multiple chronic diseases, men had higher odds of ED use (23). However, in a study assessing the ED experiences of Medicare beneficiaries, women reported a more positive experience than men in interacting with staff and receiving timely care, but they reported worse experiences than men in getting the type of care they felt they needed (24). Further studies on the relationship between sex or gender and relationship building with clinicians will provide more insight into improving shared decision-making and influencing health care use. Many studies on age, shared decision-making, and ED use in the past 10 years focused on adults aged 65 or older (25,26). Our findings showed that adults aged 18 to 39 years used the ED more than other age groups, suggesting the need for more studies assessing ED use across multiple age ranges. Future studies should assess the parameters of shared decision-making among various age groups and how beliefs or perceptions evolve.

Another interesting finding in our study was that, among those who reported a high level of shared decision-making, Hispanic-only survey respondents had lower odds of ED use than White-only survey respondents. The Hispanic-only group, overall, used the ED less than all other racial and ethnic groups in our study, with the exception of the Asian group. This finding is consistent with a scoping review study on Hispanic health that showed Hispanic adults are less likely to have visited a health care provider than all other racial or ethnic groups, possibly because of social and economic disparities, non–US-born or undocumented status, and mistrust of the health care system (27).

Among enabling factors, having public-only insurance, compared with private insurance, increased the likelihood of ED use. These findings align with other findings on insurance status and health care use. Common characteristics of Medicaid beneficiaries are more comorbidities and lower income, which are risk factors for higher rates of ED use (28). Although having insurance helps with access, Medicaid beneficiaries still experience barriers to care, such as difficulty finding medical providers that accept their insurance and lacking access to the same primary care provider over time to build a relationship and a continuous health improvement plan (28,29). Moreover, because Medicare beneficiaries are predominantly older and have more chronic diseases than non-Medicare beneficiaries, a higher rate of health care use is expected (30). Studies on innovative methodologies to improve shared decision-making among public insurance beneficiaries is needed and would be a benefit both for patients and health systems because of the possibility of further decreasing ED use.

Not surprisingly, respondents with poor self-perception of general health status, the single need factor examined in our study, had the highest rates of ED use (48.2%), while those with excellent perceptions of health status had the lowest rates of ED use (11.1%). Additionally, a high level of shared decision-making and the odds of ED use were highest among those with poor self-perception. A study on shared decision-making and medication adherence, by Milky and Thomas (10), also showed that self-perceived health status was significantly associated with shared decision-making. Other health status–related factors outside shared decision-making, such as self-efficacy and extent of comorbidities, may contribute to ED use (31). More studies on self-perception and self-efficacy in adherence to treatment plans may provide more recommendations on how to enhance shared decision-making practices.

### Limitations

Our study has several potential limitations. First, because our study was cross-sectional, only association, not causation, can be assessed. Second, because our data were self-reported, the potential for self-reporting and social desirability biases exists. Third, we used only 1 variable from the Andersen model for need factors and only 3 variables for enabling factors. Other components for need could include social determinants of health, and environmental-, policy-, and place-based factors. Fourth, an additional analysis could have been completed to assess health care providers’ knowledge of the Andersen factors and how their perceptions may have affected the success of shared decision-making, but that was not possible with this public data set. Future studies should consider additional variables that may qualify for more expansive analysis. Fifth, alternative analysis methods to assess all Andersen model factors could have been used; for example, we could have used multinomial logistical regression to assess categorical values (comparing ED use at multiple levels) rather than binary values (0 ED visits vs ≥1 ED visits). Lastly, we omitted from analysis survey respondents younger than 18 years or not diagnosed with high blood pressure, which limits the generalizability of our study. However, given the objective of the study, we believe these exclusion criteria were reasonable.

### Implications for public health

High blood pressure is a prevalent health problem in the US and worldwide. When uncontrolled, it may lead to preventable ED use and higher costs to the health system. By enhancing patient–provider communication and partnership through shared decision-making, patients may be able to improve their management of high blood pressure and not need to access emergency medical services. Health systems could consider implementing incentives for both patients and health care providers for successful chronic disease management. Future studies should expand on the perceived use of shared decision-making among people with chronic disease to improve health outcomes.

### Conclusion

Increasing shared decision-making may be an effective method for reducing avoidable ED use and improving treatment adherence. Multiple factors in addition to shared decision-making may be contributing to rates of health care service use. The Andersen model is a useful tool for considering the various factors that contribute to health care use. Future health services research can build on this study to improve the health care infrastructure at large.
